# Polyphenols from foxtail millet bran ameliorate DSS-induced colitis by remodeling gut microbiome

**DOI:** 10.3389/fnut.2022.1030744

**Published:** 2022-11-21

**Authors:** Ruipeng Yang, Shuhua Shan, Ning An, Fengming Liu, Kaili Cui, Jiangying Shi, Hanqing Li, Zhuoyu Li

**Affiliations:** ^1^Key Laboratory of Chemical Biology and Molecular Engineering of National Ministry of Education, Institute of Biotechnology, Shanxi University, Taiyuan, China; ^2^School of Life Science, Shanxi University, Taiyuan, China

**Keywords:** polyphenols, foxtail millet bran, colitis, intestinal barrier, gut microbiome

## Abstract

**Introduction:**

Polyphenols from plants possess the anti-inflammatory and gut microbiota modulated properties. Foxtail millet (*Setaria italica* L., FM) has potential medical and nutritional functions because of rich phenolic and other phytochemical components.

**Methods:**

Here, the study explored the effects of bound polyphenol of inner shell (BPIS) from FM bran on dextran sodium sulfate (DSS)-induced experimental colitis mice.

**Results:**

Results showed that BPIS administration effectively relieved the weight loss, decreased disease active index (DAI) scores, restrained the secretion of pro-inflammatory cytokines TNF-α, IL-6 and IL-1β, increased anti-inflammatory cytokines IL-10, IL-4, IL-5. BPIS prevented gut barrier damage by enhancing tight junction proteins Claudin1, ZO-1 and Occludin, increasing the number of goblet cells and facilitating the gene expressions of mucin family. In addition, BPIS restored the gut microbiota composition and increased the relative abundance of commensal bacteria such as *Lachnospiraceae* and *Rikenellaceae* and restrained the growth of *S24-7* and *Staphylococcaceae*. Concentrations of short-chain-fatty acids (SCFAs) generated by gut microbiota were elevated in BPIS treated colitis mice.

**Conclusion:**

These data suggest that BPIS effectively ameliorates DSS-induced colitis by preventing intestinal barrier damage and promoting gut microbiota community.

## Graphical Abstract

BPIS ameliorate DSS-induced colitis by remodeling gut microbiome and restoring intestinal barrier.
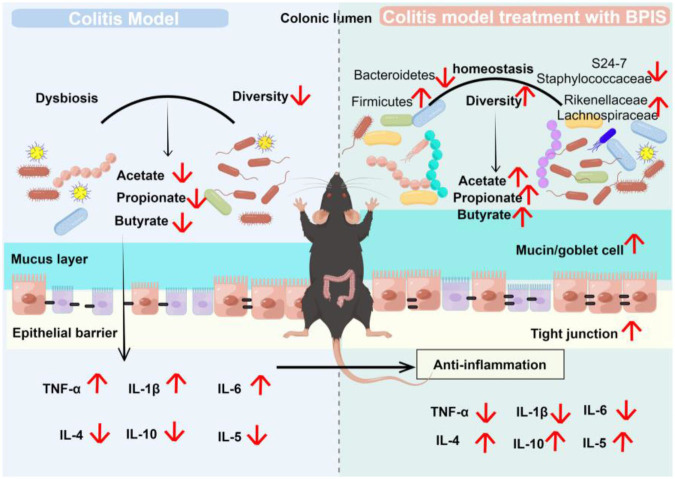

## Introduction

Foxtail millet (*Setaria italica* L.), as one of the oldest cultivated grain crops, originated in North China and is an indispensable food crop in the human diet worldwide (1). FM bran has been reported to possess antioxidant, antitumor, and immunomodulatory properties, which may account for its richness in multiple nutritious elements including protein, lipid, dietary fiber, phytosterol, polyphenols, γ-oryzanol, and squalene (2). During processing, foxtail millet is dehulled, milled, and polished, producing a by-product called coarse bran.

Daily consumption of foods rich in polyphenols, such as green tea, vegetables, and grains, has been linked to a reduced risk of inflammatory bowel disease (IBD) (3, 4). IBD is an inflammatory disease of the digestive tract, which includes Crohn's disease (CD) and ulcerative colitis (UC) (5). Despite immense research progress, the treatment of IBD remains a challenge for medical care specialists. It is characterized by loss of appetite, abdominal pain, diarrhea, and blood in the stool (6). The intestinal epithelial barrier dysfunction and the structural changes in gut microbes are considered to be the main factors in the progression and development of IBD (7). Physicians and nutritionists are making efforts to mitigate or even heal IBD with polyphenol supplements. Research performed by Boussenna et al. proved that providing polyphenol-rich preparations to ulcerative colitis mice relieves inflammation (8). Polyphenols derived from grape, apple, and barley leaves also have a positive effect on the inflammatory process (9, 10).

To exploit the potential medical and nutritional value, the present study extracted bound polyphenol of inner shell (BPIS) from FM bran and investigated the ameliorative effects of BPIS on colitis. Using DSS-induced C57BL/6J mice, it was found that BPIS supplementation effectively alleviated experimental colitis and mucosal barrier dysfunction. The results further demonstrated that BPIS restored the gut microbiota composition and significantly elevated main short-chain fatty acid (SCFA) concentrations, which were closely correlated to the improvement of experimental colitis. These findings emphasize the developed potential of BPIS as a safe and efficient agent adjuvant for IBD improvement. Supplementation of BPIS as an alternative therapy may not only address the side effects of conventional therapies but also improve the quality of life and health of individuals without compromise.

## Materials and methods

### Extraction and component detection of BPIS

The extraction process of BPIS was performed according to our previous report with some modifications (11). Briefly, foxtail millet bran (Qin-zhou-huang Millet Co., Ltd., Shanxi, China) was weighed and mixed with an acetone solution (acetone: double distilled water = 4:1) for 2 h and then centrifuged at 1,157 g for 10 min. The deposit was mixed with 2 M NaOH for 1 h and then adjusted to pH = 7. The neutralized mixture was further extracted with equivalent ethyl acetate 5 times to remove lipids. After completing centrifugation, we collected the supernatants and kept them at 45°C for evaporattion; then, the powder was vacuum freeze-dried and stored in a freezer at −80°C. To determine the composition of BPIS, the sample powder was dissolved in methyl alcohol and centrifuged for 30 min at 10,000 rpm/min. The supernatant was analyzed by UPLC-Triple-TOF/MS system (Waters Corp., Milford, MA).

### Mouse models

In this study, 6–8-week-old male healthy mice (C57BL/6J) were used (GemPharmatech Co., Ltd., Nanjing, China). All mice were kept in specific-pathogen-free (SPF) feeding rooms in the Laboratory Animal Center and Animal Laboratory of Nephrology, Shanxi Provincial People's Hospital (Shanxi, China) at a temperature of 24 ± 2°C and a humidity of 40–70% and under 24 h light-dark cycle condition. Mice were fed with sterile food and water, and autoclaved bedding litter in the cage was changed two times a week. Under the permission of the Committee on the Ethics of Animal Experiments of Shanxi University (Shanxi, China), all experiments were performed in accordance with published National Institutes of Health guidelines.

### DSS colitis and assessment of disease active index

**Figure 1:**
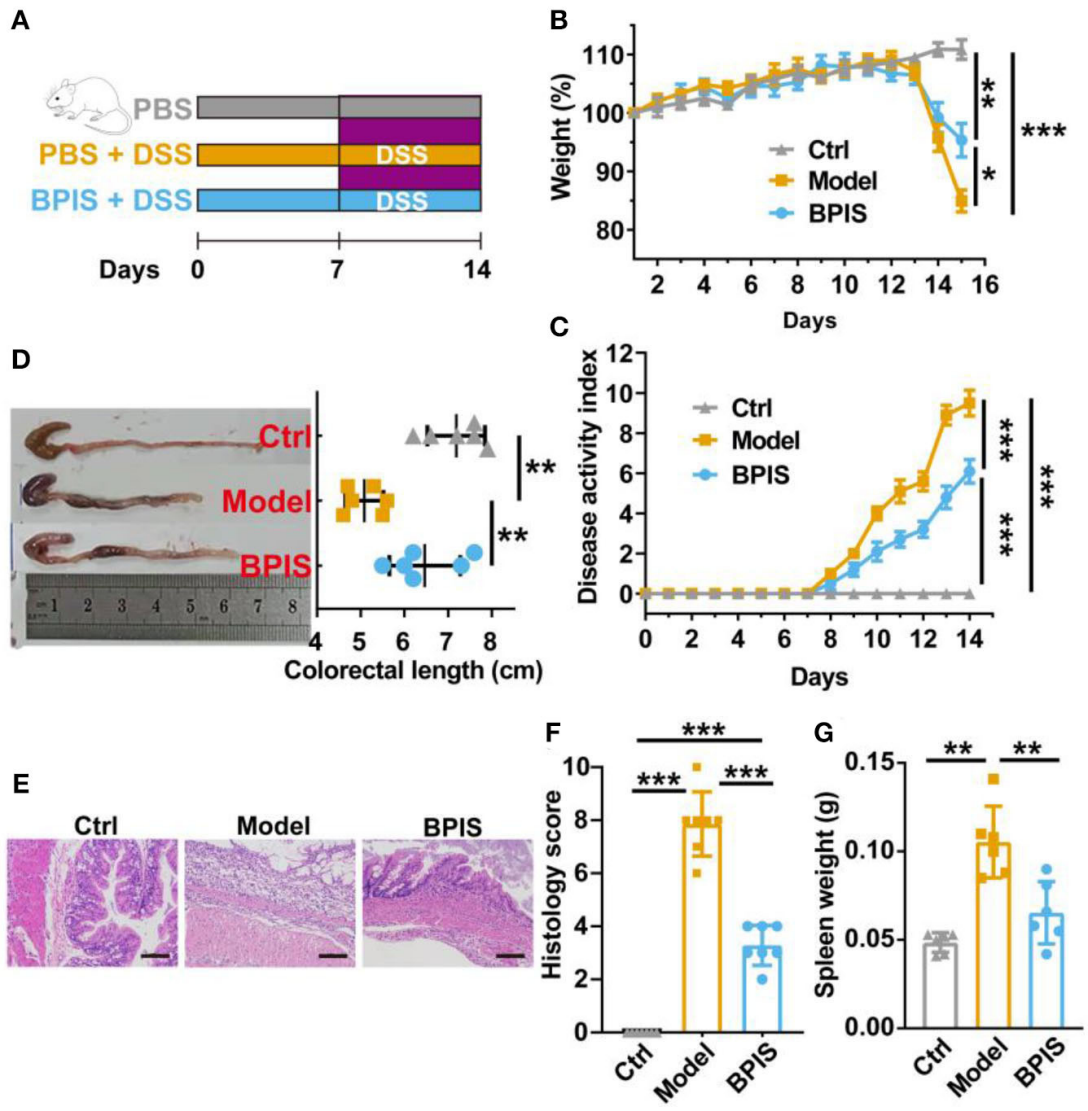
BPIS relieves dextran sodium sulfate (DSS)-induced colitis. Mice were gavaged with bound polyphenol of the inner shell (BPIS) extracted from foxtail millet bran for 2 weeks. Colitis was induced by administering 3% DSS dissolved in drinking water for 7 days. **(A)** Study design of the *in vivo* mouse experiment. **(B,C)** Percentage changes in body weight, and disease activity scores (DAI). **(D)** Colon lengths in differential groups and representative images of mice colons. **(E,F)** Representative hematoxylin and eosin (H&E)-stained histologic images, and comprehensive histology scores of the colon of experimental mice. Scale bars, 100 μm. **(G)** Spleen weight. Data are presented as the means ± SD (n ≥ 5) and were analyzed by an ordinary one-way ANOVA with Tukey's multiple comparisons. *p < 0.05, **p < 0.01, ***p < 0.001.

After a week of acclimatizing, mice were administered 3% DSS (Meilun, Cat#: M0705A) in drinking water for 7 days to induce acute colitis (Figure 1A). Mice weight, stool characteristics, and blood in feces were measured every day. The DAI index was calculated by adding the scores of percentage weight loss, stool consistency, and fecal occult blood (12). Comprehensive scores were as follows: Weight changed: 0, no loss; 1, 1–5% descend; 2, 5–10% descend; and 3, 10–15% descend; stool consistency: 0, no diarrhea; 1, loose stool; and 2, liquid stool; and fecal bleeding: 0, no bleeding; 2, moderate; 4, severe bleeding.

### Histology

Colon tissues were fixed with 4% paraformaldehyde for 24 h, embedded in paraffin, sectioned at 4 μm, and stored at room temperature for the next operation. For H&E staining, slides were sequentially stained with hematoxylin solution and eosin dye. For alcian blue periodic acid–Schiff (AB-PAS) staining, sections were stained using the AB-PAS Stain Kit (Servicebio, Wuhan, China). Stained slides were inspected and photographed under the inverted microscope. The degree of inflammatory lesions was assessed using the following 0–4 point scale (13): 0, none; 1, mild; 2, moderate; and 3, severe. The level of inflammation infiltration scale is as follows: 0, none; 1, mucosa; 2, mucosa and submucosa; and 3, transmural. The gradation of the epithelial/crypt damage scale is as follows: 0, none; 1, basal 1/3; 2, basal 2/3; 3, crypt loss; and 4, crypt loss and tissue epithelial damage. The histology score is the sum of the above indices.

### RNA isolation, cDNA synthesis, and qRT-PCR

Analysis of the mRNA expression levels was performed according to a previous report with some modifications (11). Briefly, colonic tissues were homogenized using an automatic freeze grinder (Jing Xin, Shanghai, China). Total RNA was isolated using RNAiso Plus (Takara, Japan) and quantified at 500 ng for reverse transcription synthesis of cDNA using the HiScript II Q RT SuperMix kit (Vazyme, Nanjing, China) according to the manufacturer's protocol. The sequences of primers are listed in Table 1. Relative mRNA expressions of genes were normalized to *Gapdh*.

**Table 1:** Primer design of qPCR.

**Gene**	**Sequences (5^′^to 3^′^)**
	
*Tnf-α*	Forward primer: CGCTCTTCTGTCTACTGAACTTCGG
	Reverse primer: GTGGTTTGTGAGTGTGAGGGTCTG
*Il-1β*	Forward primer: CACTACAGGCTCCGAGATGAACAAC
	Reverse primer: TGTCGTTGCTTGGTTCTCCTTGTAC
*Il-6*	Forward primer: CTTCTTGGGACTGATGCTGGTGAC
	Reverse primer: AGTGGTATCCTCTGTGAAGTCTCCTC
*Il-33*	Forward primer: AGACCAGGTGCTACTACGCTACTATG
	Reverse primer: ACTCATGTTCACCATCAGCTTCTTCC
*Il-10*	Forward primer: CTGGACAACATACTGCTAACCGACTC
	Reverse primer: ACTGGATCATTTCCGATAAGGCTTGG
*Tff3*	Forward primer: CCTGGTTGCTGGGTCCTCTG
	Reverse primer: GCCACGGTTGTTACACTGCTC
*Klf4*	Forward primer: GTGCCCCGACTAACCGTTG
	Reverse primer: GTCGTTGAACTCCTCGGTCT
*Muc1*	Forward primer: GCAGTCCTCAGTGGCACCTC
	Reverse primer: CACCGTGGGCTACTGGAGAG
*Muc2*	Forward primer: GCTGACGAGTGGTTGGTGAATG
	Reverse primer: GATGAGGTGGCAGACAGGAGAC
*Muc3*	Forward primer: CGTGGTCAACTGCGAGAATGG
	Reverse primer: CGGCTCTATCTCTACGCTCTCC
*Muc4*	Forward primer: CAGCAGCCAGTGGGGACAG
	Reverse primer: CTCAGACACAGCCAGGGAACTC
*Cldn1*	Forward primer: GCTGGGTTTCATCCTGGCTTCTC
	Reverse primer: CCTGAGCGGTCACGATGTTGTC
*Ocln*	Forward primer: TGGCTATGGAGGCGGCTATGG
	Reverse primer: AAGGAAGCGATGAAGCAGAAGG
*Zo1*	Forward primer: CCACCTCGCACGCATCACAG
	Reverse primer: TGGTCCTTCACCTCTGAGCACTAC
*Gapdh*	Forward primer: ACCCACTCCTCCACCTTTGA
	Reverse primer: CTGTTGCTGTAGCCAAATTCGT

### Enzyme-linked immunosorbent assay

After the administration of DSS for 7 days, the blood sample was collected from the mouse orbital sinus (14). After standing for 1 h, blood samples were centrifuged, and serums were collected and stored in a freezer at −80°C. To detect the levels of cytokines in mice serums, ELISA kits (Andy Gene Biotechnology Co., Ltd., Beijing, China) of TNF-α, IL-1β, IL-6, IL-10, IL-4, and IL-5 were used according to the manufacturer's guidance.

### Immunofluorescence staining

An immunofluorescence assay for Claudin-1 (Servicebio, Cat#: GB11032), Occludin (Servicebio, Cat#: GB111401), and ZO-1 (Servicebio, Cat#: GB111981) was provided by Servicebio Technology Co., Ltd. (Wuhan, China). For immunofluorescence, slides were stained by antibodies following microscopy detection, and then, the represented images were collected. All figures were analyzed and quantified using Image-Pro Plus 6.0.

### Microbial sequence and analysis

The sequencing service was provided by Personalbio Technology Co., Ltd. (Shanghai, China). Stool samples were collected on day 8 of DSS administration for genomic DNA extraction using the Omega Soil DNA Kit (D5625-01) (Omega Bio-Tek, Norcross, GA, USA) according to the manufacturer's guidance. DNA was amplified by PCR using the Q5 High-Fidelity DNA Polymerase (NEB, USA) using V3–V4 region primers (forward, 5′-ACTCCTACGGGAGGCAGCA-3′; reverse, 5′-CGGACTACHVGGGTWTCTAAT-3′). PCR products were purified, quantified, and sequenced using the Illumina MiSeq platform and MiSeq Reagent Kit version 3. The microbiota analysis was performed using QIIME2 and R software (15).

### SCFA analysis in colonic contents

The mice for SCFA analysis wwere provided by Biotree Biomedical Technology Co., Ltd. (Shanghai, China). Briefly, after collecting stools and serums, mice were sacrificed and the colon tissues were cut off and quickly frozen in liquid nitrogen; then, they were transferred to a freezer at −80°C. For analyzing the concentrations of SCFAs, colons were taken out from −80°C, and the contents were carefully extruded with sterile forceps on ice. The contents combined with 1 ml H_2_O were added into 2 ml EP tubes for vortexing for 10 s. The mixture was then transferred to a ball mill, crushed at 40 Hz for 4 min until it homogenized, and then sonicated in ice water for 5 min, and the process was repeated three times. After centrifuging (20 min, 5,000 rpm, 4°C), the supernatant was transferred into a fresh EP tube. Next, 0.1 ml of 50% H_2_SO_4_ and 0.8 ml of extracting solution were added as internal standards, and the supernatant underwent further vortexing, oscillation, and ultrasound treatment in ice water for 10 min. After centrifuging (15 min, 10,000 rpm, 4°C), the supernatant was moved into a new 2 ml glass tube for gas chromatography-mass spectrometry (SHIMADZU GC2030-QP2020 NX) analysis. The detection uses an HP-FFAP capillary column (Agilent Technologies, Inc., USA). Notably, 1 μl of the analyte was injected in split mode (5:1). Helium was used as the carrier gas, the front inlet purge flow was 3 ml/min, and the gas flow rate through the column was 1 ml/min. The initial temperature was kept at 80°C for 1 min, then raised to 200°C at a rate of 10°C min^−1^ for 5 min, then kept at 240°C for 1 min at a rate of 40°C min^−1^. The injection, transfer line, quad, and ion source temperatures were 240, 240, 150, and 200°C. The energy was −70 eV in electron impact mode. The mass spectrometry data were acquired in scan/SIM mode with the m/z range of 33–150 after a solvent delay of 3.5 min.

### Statistical analysis

Statistical analysis was carried out using SPSS 24 (SPSS Inc., USA) and the R project (version 4.1.2) with RStudio (version 1.1.463). Statistical significances between the two groups were tested by a two-tailed, unpaired Student's *t*-test. A one-way analysis of variance (ANOVA) followed by Tukey's multiple comparisons test was used for more than two groups. The results were represented as means ± SD. A *p*-value of < 0.05 was deemed statistically significant (^*^p < 0.05, ^**^p < 0.01, ^***^p < 0.001).

## Results

### BPIS relieves acute colitis in mice

BPIS contains 12 phenolic acids (Table 2). To evaluate the consequences of BPIS on inflammatory bowel disease, we administered mice with 3% DSS water to induce an IBD model. Based on the weight of every mouse, 75 mg/kg BPIS was administered by gavage to evaluate the improvement in colitis (Figure 1A). After 3% DSS water was given, the body weight gradually decreased and dropped to 85% on day 8 (Figure 1B). Meanwhile, DAI scores of comprehensive stool consistency, bloody stool, and weight loss also displayed a consistent trend (Figure 1C). Moreover, DSS-induced colitis mice showed shorter colon length (Figure 1D) and splenomegaly (Figure 1G), suggesting that DSS-induced colon inflammation might be spread to the whole body. Therefore, we suspect whether DSS devastated the structure of the colon. H&E staining showed that the proximal colons of model mice were histologically damaged, such as epidermis structure loss, epithelial barrier disruption, and distinct inflammatory cell involvement (Figures 1E,F). Nevertheless, BPIS (75 mg/kg) supplementation effectively reduced the weight and improved the DAI scoresbesides relieving colon shortening and splenomegaly and attenuating histological injuries (Figures 1B–G). These data demonstrate that BPIS treatment significantly ameliorates acute inflammation in DSS-induced mice.

**Table 2:** Twelve phenolic acids in bound polyphenol of the inner shell (BPIS) extracted from millet bran (MW, molecular weight).

**No**.	**BPIS components**	**Molecular formula**	**MW**
1	4-Hydroxybenzoic acid	C_7_H_6_O_3_	138
2	p-Coumaric acid	C_9_H_8_O_3_	166
3	Vanillic acid	C_8_H_8_O_4_	168
4	Ferulic acid	C_10_H_10_O_4_	194
5	Isoferulic acid	C_10_H_10_O_4_	194
6	Syringic acid	C_9_H_10_O_5_	198
7	Vanillic acid 4-O-β-D-glucopyranoside	C_14_H_18_O_9_	330
8	Ferulic acid 4-O-β-D-glucopyranoside	C_16_H_20_O_9_	356
9	Glucosyringic acid	C_15_H_20_O_10_	360
10	4,4′-dihydroxy-3,5′-dimethoxy,3′-bicinnamic acid	C_20_H_18_O_8_	386
11	Biferulic acid	C_20_H_18_O_9_	402
12	Vitexin	C_21_H_20_O_10_	432

### BPIS suppresses the expression and secretion of inflammatory cytokines

**Figure 2:**
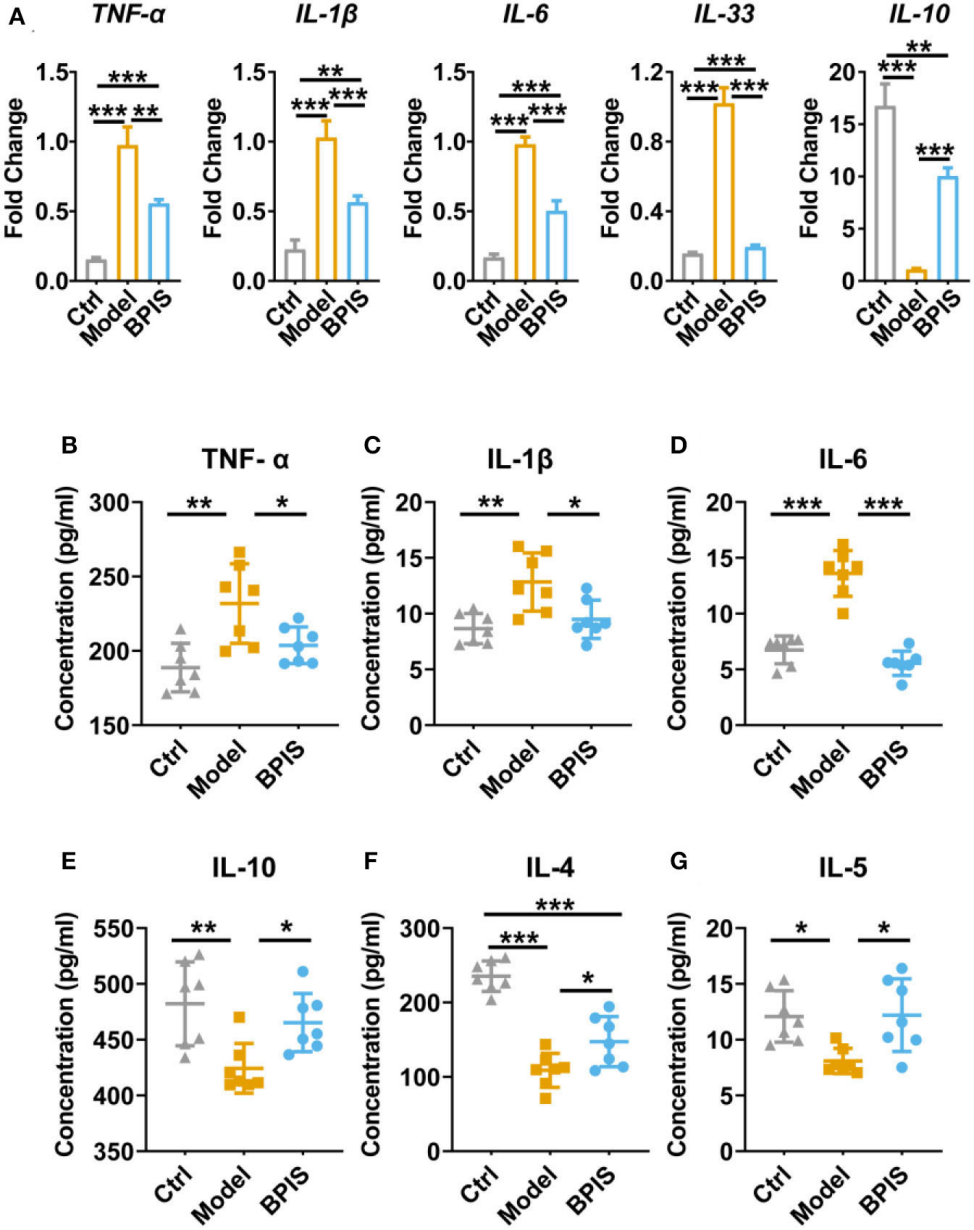
BPIS suppressed inflammatory cytokines *in vivo*. **(A)** mRNA expressions of inflammatory cytokines including *Tnf-*α*, Il-1*β*, Il-6, Il-33*, and *Il-10* in colon tissues. **(B–G)** Concentrations of the cytokines TNF-α **(B)**, IL-6 **(C)**, and IL-1β **(D)**; Treg-type cytokine IL-10 **(E)**, and Th2-type cytokines IL-4 **(F)** and IL-5 **(G)** were determined by ELISA kit. Data are presented as the means ± SD (*n* ≥ 5) and were analyzed by an ordinary one-way ANOVA with Tukey's multiple comparisons. *p < 0.05, **p < 0.01, ***p < 0.001.

In the present study, as inflammation infiltration and colon tissue damage were found, we next evaluated typical pro-inflammatory and anti-inflammatory cytokines. First, qPCR data indicated that the mRNA expressions of cytokines, including TNF-α, Il-1β, Il-6, and Il-33, were significantly diminished in colons from the BPIS-treated group, while the gene level of IL-10 was increased up to 10-folds as compared to those of the model group (Figure 2A). Moreover, to monitor the secretory proteins related to colitis, we assessed the concentrations of cytokines in mouse serum using an ELISA kit, and the results showed that BPIS treatment significantly reduced the level of inflammatory factors TNF-α, IL-1β, and IL-6 (Figures 2B–D) but elevated the level of the Treg-type cytokines IL-10 (Figure 2E), Th2-type cytokines IL-4 (Figure 2F), and IL-5 (Figure 2G). These findings suggest that BPIS suppresses the secretion of pro-inflammatory factors and thus mitigates mice colitis.

### BPIS ameliorates DSS-induced mucus disruption and tight junction depletion

**Figure 3:**
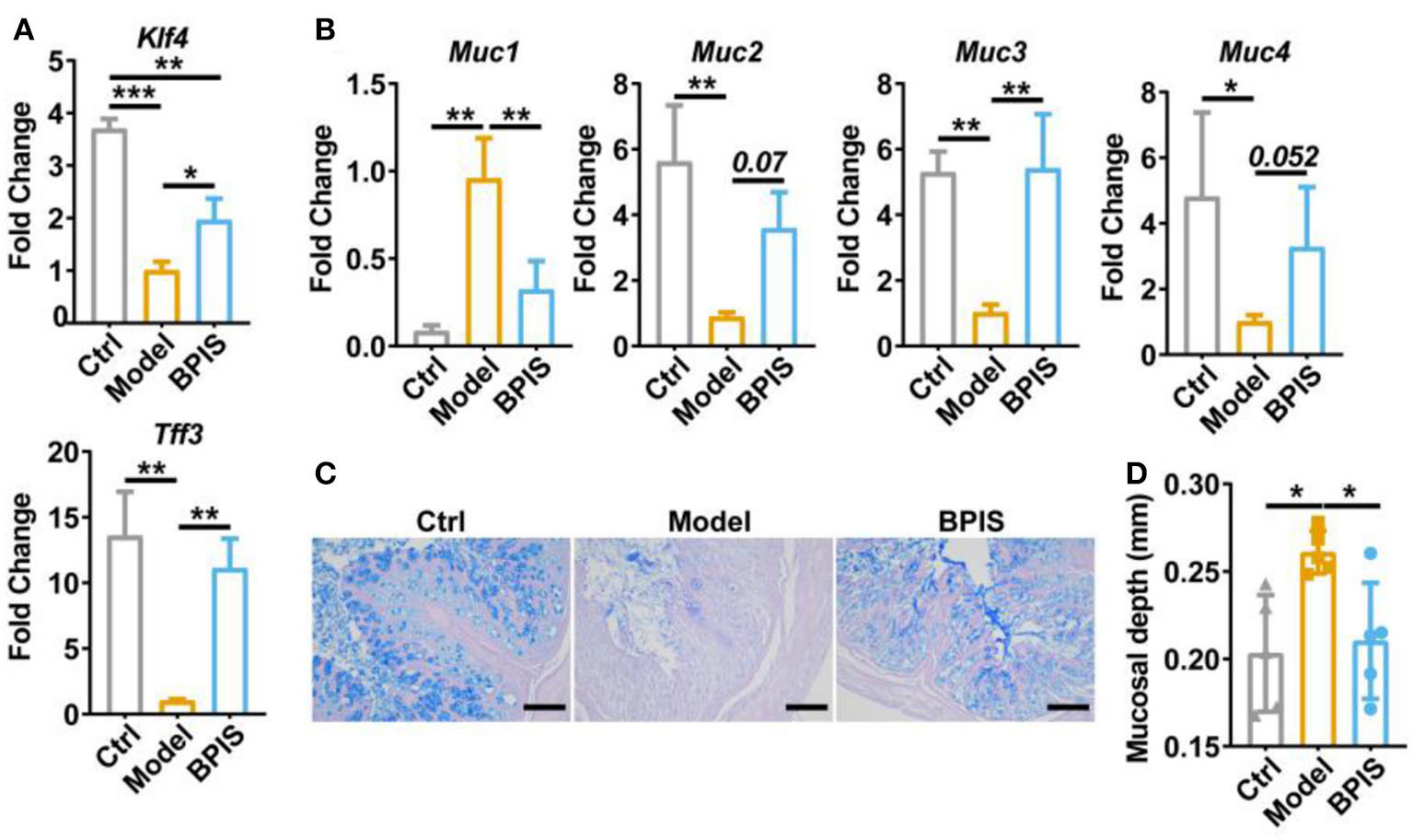
BPIS ameliorated mucus disruption and goblet cell depletion. **(A,B)**, qPCR analysis for expression of goblet cell maturation markers *Tff3* and *Klf4* **(A)** and mucin genes *Muc1, Muc2, Muc3*, and *Muc4* **(B)**. **(C)** Representative images of alcian blue-stained colonic sections, Scale bars, 100 μm. **(D)** Muscular layer width in the colon of mice was quantified. Data are presented as the means ± SD (*n* ≥ 5) and were analyzed by an ordinary one-way ANOVA with Tukey's multiple comparisons. *p < 0.05, **p < 0.01, ***p < 0.001.

**Figure 4:**
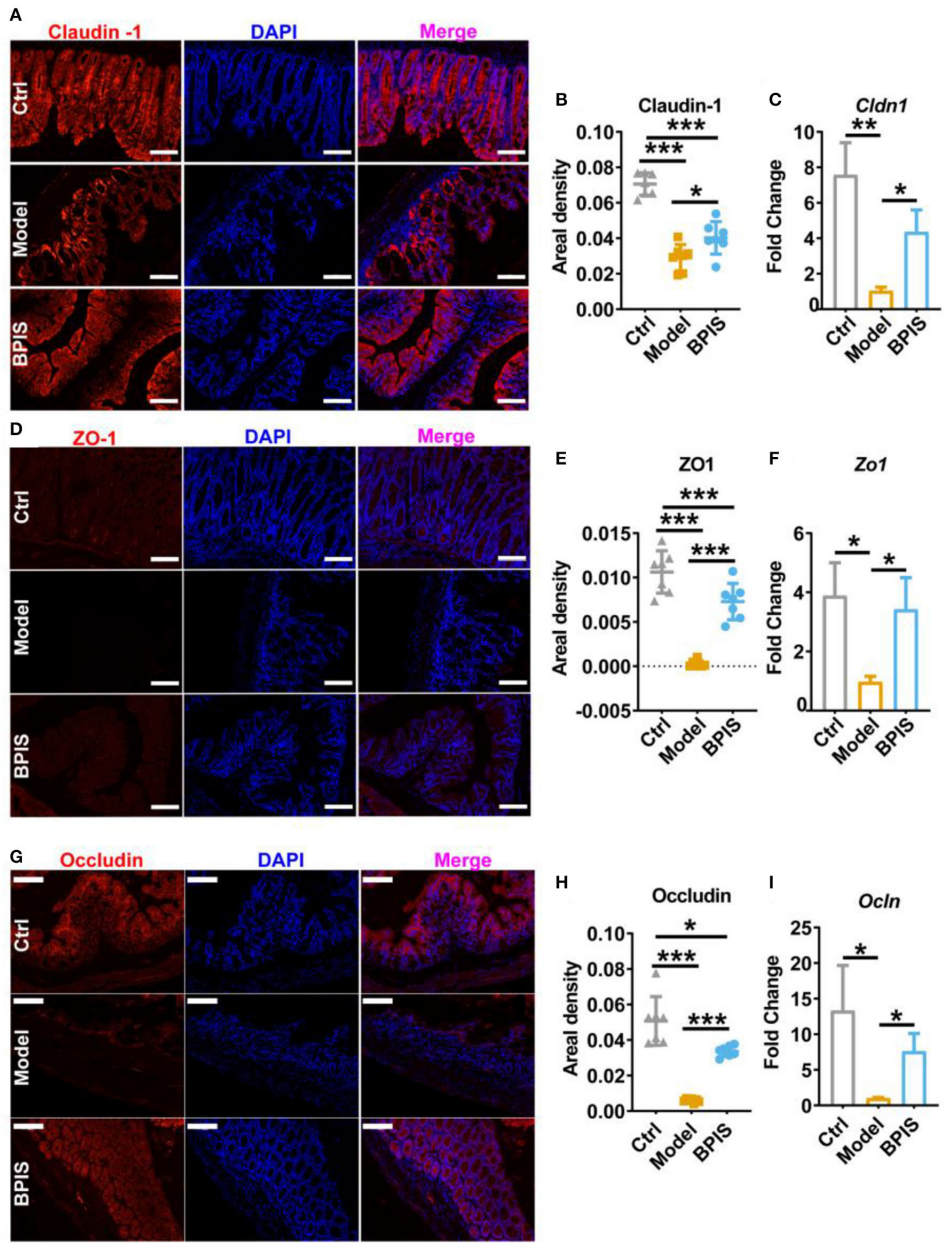
BPIS inhibited the loss of epithelial barrier integrity. Representative immunofluorescence staining images of tight junction proteins Claudin-1 **(A)**, ZO-1 **(D)**, and Occludin **(G)**, and the positive areal density **(B,E,H)** and the mRNA expression encoding these proteins in colon tissues. **(C,F,I)**. Scale bars, 100 μm. Data are presented as the means ± SD (*n* ≥ 5) and were analyzed by an ordinary one-way ANOVA with Tukey's multiple comparisons. *p < 0.05, **p < 0.01, ***p < 0.001.

Intestinal barrier dysfunction leads to increased severity in patients with IBD, which has been demonstrated in animal models (16, 17). Throughout the gut, mucus-secreting goblet cells form a mucosal barrier that prevents the invasion of pathogenic bacteria and preserves a near-sterile epithelium (18). *Tff3* and *Klf4* have been reported to act as markers of goblet cell maturation. Proteins Muc1, Muc2, Muc3, and Muc4 are members of the mucin family. qRT-PCR analysis showed that BPIS treatment upgraded the gene expression levels of *Tff3, Klf4* (Figure 3A), *Muc2, Muc3*, and *Muc4* (Figure 3B), which manifested the facilitation of BPIS on the mucosal barrier in IBD mice. Of note, the gene expression encoding *Muc1* was upgraded in DSS-induced colitis in the mouse colon and was significantly restrained by BPIS. A previous study proved that *Muc1* acts as a tumor-related factor and is abnormally expressed in patients with IBD (19). Moreover, AB-PAS staining displayed a substantial elevation in mucus-producing goblet cells in the colon of BPIS gavage mice compared with that of untreated colitis mice (Figure 3C). Investigation of colonic morphology demonstrated that BPIS supplementary colitis mice exhibited a clear increase mucosal depth compared with the model group (Figure 3D). Furthermore, tight junction proteins, including Claudin-1, ZO-1, and Occludin, were detected. Immunofluorescence images showed that tight junction proteins were significantly inhibited in inflammatory mice colon, while their increased levels were observed after BPIS gavage (Figures 4A–H). Consistently, the gene expressions of *Cldn1* (Figure 4C), *Zo-1* (Figure 4F), and *Ocln* (Figure 4I) in mouse colon tissues were upregulated by BPIS. Overall, these results indicate that BPIS maintained intestinal barrier dysfunction in inflammatory mice, including the intestinal mucosal barrier and the intestinal epithelial barrier.

### Treatment with BPIS increases the diversity of gut microbiota

**Figure 5:**
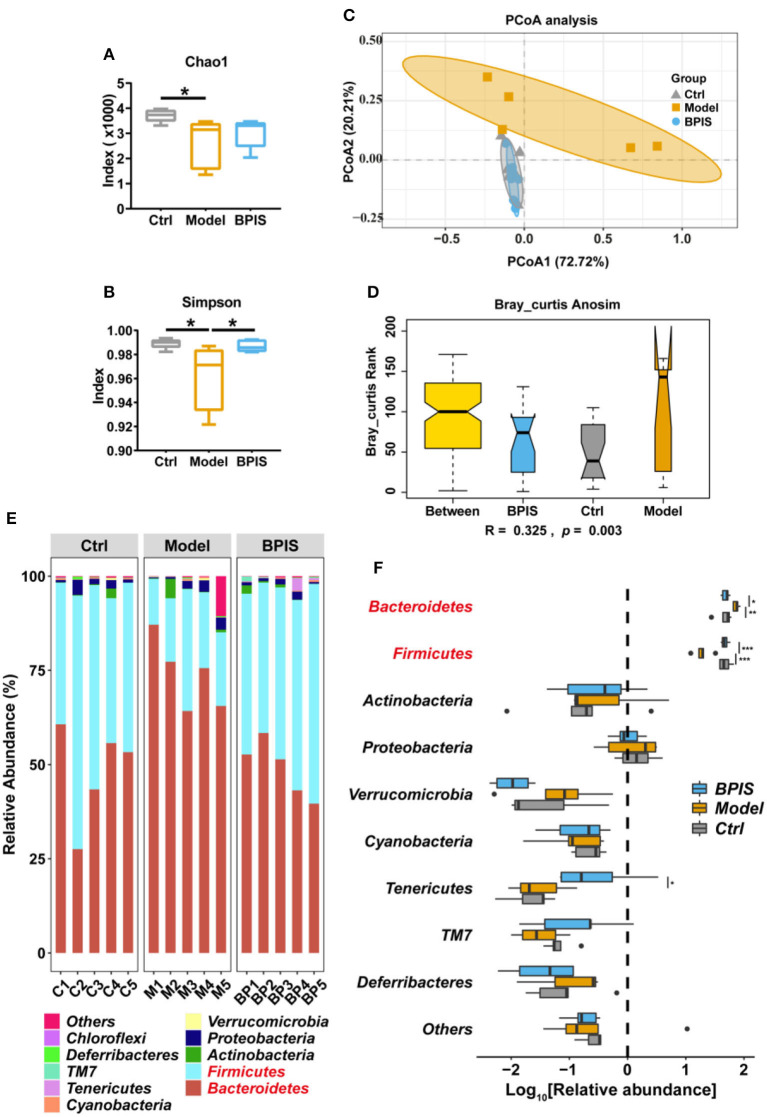
BPIS increased microbial diversity and reshaped the microbial community. Stool samples from different groups were used for microbiome analysis. **(A,B)** Alpha diversity analysis of gut bacterial richness **(A)** and diversity **(B)** from different mouse groups. **(C)** Principal coordinates analysis (PCoA) plot of the gut microbiota composition from different groups. **(D)** Bray_curtis ANOISM analysis. Boxes are the interquartile range; median values are bands within the boxes; whiskers are 1.5 times the IQR. **(E)** Taxonomic distributions of the relative abundances at the phylum level. **(F)** Taxonomic composition indices on a log_10_ scale for each phylum of fecal bacteria from mice. Data are presented as the means ± SD (*n* ≥ 5) and were analyzed by an ordinary one-way ANOVA with Tukey's multiple comparisons. *p < 0.05, **p < 0.01, ***p < 0.001.

**Figure 6:**
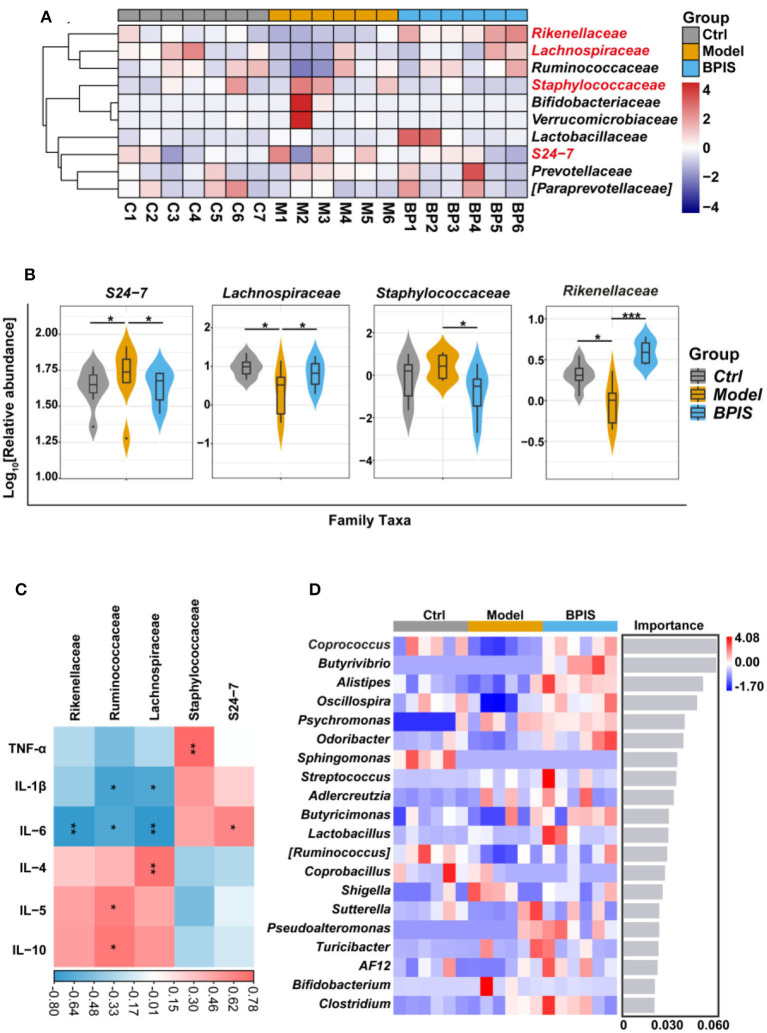
BPIS regulated the growth of certain commensal bacteria at the family level. **(A)** Heatmap showing the relative abundance of families, ranking the top 10 from each sample. **(B)** Taxonomic composition indices on a log_10_ scale for significantly different families of fecal bacteria from differential groups. **(C)** Correlation analysis between different families and inflammatory cytokines in DSS-induced colitis mice. **(D)** At the genus level, random forest analysis and cross-validations were performed on the absolute abundance of microbial species in each group of samples. Data are presented as the means ± SD (*n* ≥ 5) and were analyzed by an ordinary one-way ANOVA with Tukey's multiple comparisons. *p < 0.05, **p < 0.01, ***p < 0.001.

After treating intestinal barrier damage and inflammation in the mice model with DSS, we further investigated the influence of BPIS on the gut microbiota composition of DSS-induced inflammatory mice. Mice stools were analyzed for 16S rRNA sequencing on day 8 of DSS administration. The results showed that, although microbial richness (Figure 5A) was not restored by BPIS, the gut microbiota diversity (Figure 5B) was significantly increased as compared to the PBS-treated mice. Furthermore, the alteration of microbial construction was examined by the principal coordinates analysis (PCoA), and Figure 5C shows a marked structural shift in samples of the model group in contrast to those of the control and BPIS groups. Analysis of similarities (ANOSIM) further confirmed the microbiota structure remodeling by BPIS (Figure 5D). At the phylum level, the most abundant were *Firmicutes* and *Bacteroidetes*, and BPIS remarkably expanded the relative abundance of *Firmicutes* but suppressed the growth of *Bacteroidetes* (Figures 5E,F). At the family level, BPIS significantly enriched *Lachnospiraceae* and *Rikenellaceae*, whereas it decreased *S24-7* and *Staphylococcaceae* (Figures 6A,B). Spearman's rank correlation showed the correlation between different families and cytokines (Figure 6C). Random forest analysis was performed to identify the specific bacterium that was responsible for improving colitis and remodulating the microbiota community. As shown in Figure 6D, two genera *Coprococcus* and *Butyrivibrio* belonging to *Lachnospiraceae* and *Alistipes* belonging to *Rikenellaceae* are important performers involved in the BPIS remodeling microbiota structure.

### Short-chain fatty acids were increased by BPIS treatment

**Figure 7:**
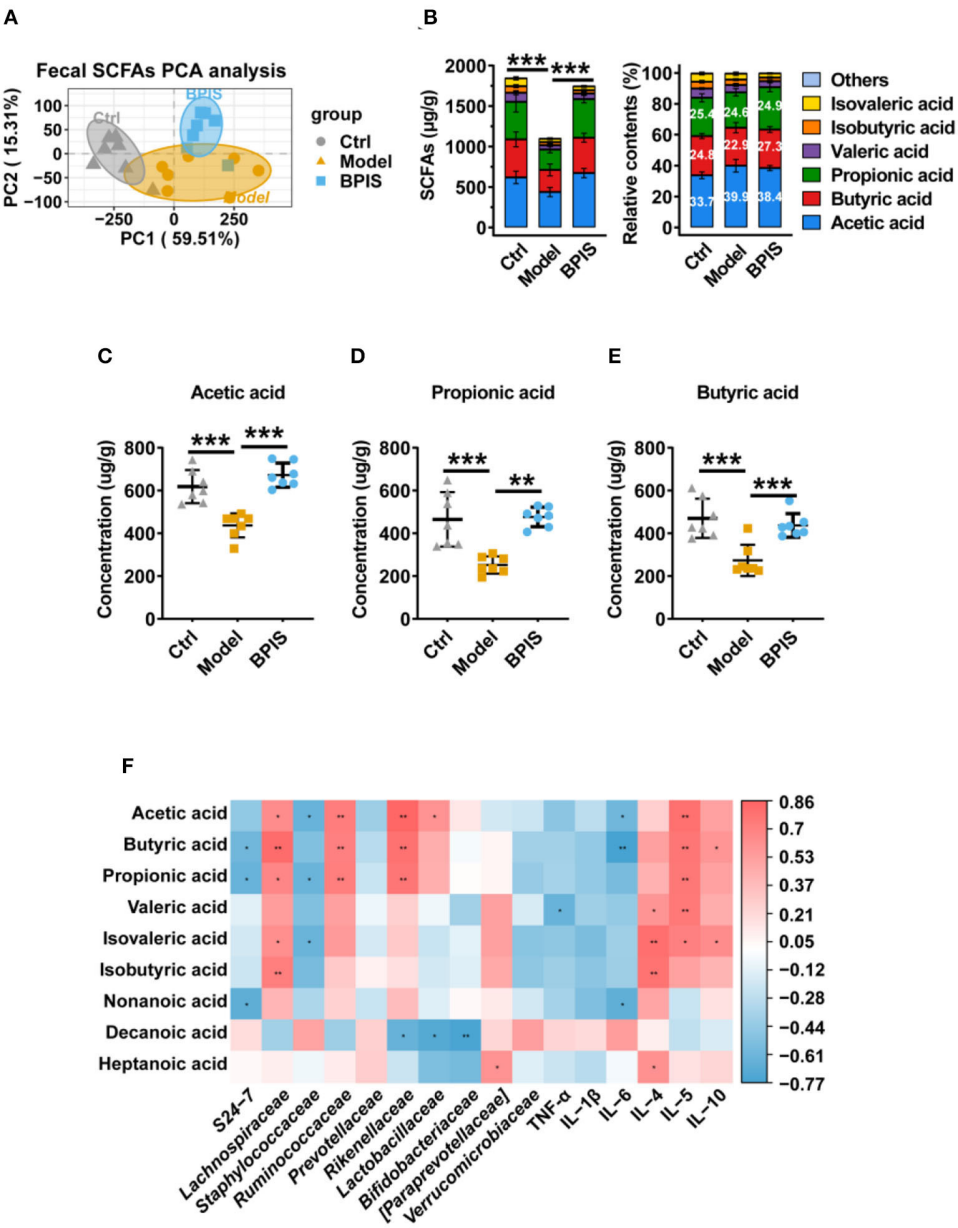
Effects of BPIS on fecal levels of SCFAs in DSS-colitis mice. **(A)** The absolute contents of SCFAs in the proximal colon from DSS-induced and BPIS*-*gavage-DSS-induced samples were used for principal components analysis. **(B)** The absolute and relative contents of SCFAs in the proximal colon from mice samples were sequentially collected and were detected using high-resolution gas chromatography and mass spectrometry (GC-MS). **(C–E)** The concentrations of acetic acid, propionic acid, and butyric acid. **(F)** Correlation analysis among gut microbiota, inflammatory cytokines, and colonic SCFAs. Data are presented as the means ± SD (*n* ≥ 5) and were analyzed by an ordinary one-way ANOVA with Tukey's multiple comparisons. *p < 0.05, **p < 0.01, ***p < 0.001.

As SCFAs are one of the main metabolites produced by commensal microorganisms, we analyzed the SCFA contents in mice colons. As shown in Figure 7A, the PCA showed a distinct separation between the 2 groups. Total SCFAs were significantly elevated by BPIS; nevertheless, the type and the relative contents of SCFAs were not changed after BPIS treatment (Figure 7B). Among the main SCFAs, treatment with BPIS markedly increased the levels of acetic acid, propionic acid, and butyric acid compared with DSS-induced mice (Figures 7C–E). To find the relationship between microbiota and fecal SCFAs in experimental colitis mice, we selected Spearman's correlation analysis. The results showed that there were notable correlations between the SCFA levels and microbes. The families *Lachnospiraceae, Ruminococcaceae*, and *Rikenellaceae*, enriched by BPIS treatment, had been discovered to have significant positive correlations with three main SCFAs, while *S24-7* was negatively correlated with propionate and butyrate, and the family *Staphylococcaceae* showed negative correlation with acetate and propionate. In addition, analysis of the correlation between SCFAs and cytokines also found that cytokines TNF-α, Il-1β, and Il-6 were negatively correlated, while cytokines IL-4, IL-5, and IL-10 were positively correlated with SCFAs (Figure 7F).

## Discussion

Given the substantial economic cost and the side effects such as destroying the host intestinal microbial balance in conventional drug-based therapies, it is still urgent to develop new effective and low-toxic therapeutic strategies for colitis (20). Recently, natural products exhibited efficient potential for IBD in experimental models and clinical trials (21). Accumulating studies proved that polyphenol supplementations such as quercetin (22), curcumin (23), and resveratrol (24) have beneficial effects on gut microbiota restoration and therapy for patients with IBD. Foxtail millet, as one of the oldest cultivated grain crops, has been conveyed to possess possible therapeutic and nutritional values such as antioxidant, antitumor, and anti-inflammatory activities. Although FM bran is a by-product of FM processing into millet, it is rich in nutrients and phytochemicals, which may endow it with physiological activities. BPIS was extracted from FM bran, which was traditionally seen as feed for livestock or returned to the field. The present study exploited the potential medicinal value of BPIS in DSS-induced colitis. These findings may lead us to conduct research and develop more economical, green, yet safe, and efficient adjuvant, therapeutic agents using FM bran as the raw material for IBD.

Pro-inflammatory cytokines are crucial in the pathogenesis of IBD. TNF-α, which is oversecreted by innate cells, is a pro-inflammatory factor in the lamina propria of patients with IBD (25). IL-1β is a classic pro-inflammatory cytokine known to amplify innate immune responses (26). Studies have been exploring the potential to develop anti-IL-6 agents for IBD treatment (27). IL-10 plays a critical role in maintaining the regulatory phenotype and preventing the development of IBD (28). IL-4 and IL-5, two Th2 cytokines, are potent anti-inflammatory factors and participate in protecting against infections and keeping tissues from the potential collateral injury resulting from inflammation (29, 30). In mice colons, TNF-α, IL-1β, and IL-6 were restrained, whereas the Treg-type cytokine IL-10 and Th2-type cytokines, IL-4 and IL-5, were increased by BPIS treatment (Figure 2). Of note, it is not clear how BPIS regulates the intestinal immune system to affect the secretion of inflammatory factors and whether it can be used as a supplement for immune enhancement, which will be one of the contents we need to explore in the future. Patients with IBD display several functional defects in the mucosal layer composition and the adhesion molecules that regulate paracellular permeability (31). In some types of ulcerative colitis, the mucus layer becomes penetrable, increasing the risk of pathogen invasion (32). In this study, our data showed that administration of BPIS restored the intestinal mucosal function disrupted by DSS, which was proven by detecting markers of goblet cell maturation, mucin production genes, and mucin secretion levels (Figure 3). Tight junction dysfunction is another cause of “leaky gut,” which enhances inflammatory progression in patients with colitis. The tight junction is gathered by both transmembrane proteins such as Occludin, different Claudins, and peripheral membrane proteins such as ZO proteins (16). In the present study, whether at the gene or protein level, treatment with BPIS upregulated the expression of Claudin-1, Occludin, and ZO-1 in colitis mice colons (Figure 4). Although IBD is considered to be multifactorial conditions in its course, dramatic changes in the commensal microbiota are always along with the whole process (33). Studies reported that the gut microbiota of patients with IBD displayed a significant reduction in commensal microbiota diversity compared with healthy subjects (34). Among the gut microbiota, *Firmicutes* and *Bacteroidetes* are two dominant phyla, representing 90% of the whole microbiome. Interestingly, the ratio between these two phyla has been associated with maintaining homeostasis and serves as a marker in various pathologies. For instance, an increasing *Firmicutes*/*Bacteroidetes* ratio was observed in obesity, whereas a decreasing ratio was observed in patients with IBD (35). According to these studies, the data of 16S rRNA gene sequencing also disclosed that BPIS treatment elevated the relative abundance of *Firmicutes* and decreased that of *Bacteroidetes* (Figure 5). This will be an instructive vision for the research or even development of BPIS as agents for modifying the *Firmicutes*/*Bacteroidetes* ratio and treating IBD. At the family level, it was found that BPIS increased the growth of *Lachnospiraceae* and *Rikenellaceae*. The abundance of *Lachnospiraceae* has been reported to be significantly decreased in patients with IBD compared to healthy volunteers (36). *Rikenellaceae* has been reported to be protective bacteria in UC and can be enriched by natural products such as polysaccharides (37). However, BPIS decreased the relative abundance of *Staphylococcaceae*, which contains genus Staphylococcus, noted for encompassing several medically significant pathogens. These findings support the gut microbiota-recovery effects of BPIS on IBD (Figure 6). An important role of microbiome in the host intestinal system is producing SCFAs, which are predominant fuels for bowel epithelial cells (38). One characteristic of IBD is the destruction of SCFA production, which suppresses energy acquisition for colonocytes and facilitates local infiltration of mucosal inflammation (38). Recently, SCFAs are supposed to be a prospective supplement in the current clinical therapy of active patients with IBD and diversion colitis. Therefore, considering the ability of BPIS to increase concentrations of intestinal SCFAs in IBD mice (Figure 7), it may be prospective to supplement it with SCFAs as an adjuvant to improve IBD therapeutic efficiency in the future. Considering the possible application of BPIS for humans in the future, dose-equivalent conversion is an issue that must be solved. Shannon et al. reported a body surface area normalization method to translate the dose from mice to human: Human equivalent dose (mg/kg) = Mice dose (mg/kg) × (Mice *Km*/Human *Km*) (39). In this study, we administered a dose of 75 mg/kg BPIS to mice, which equates to a dose of 12.16 mg/kg in humans. Accordingly, for a 60 kg adult, the daily consumption of BPIS is approximately 729.6 mg. Conventionally, FM bran is the by-product from foxtail millet to polished grain and was deemed an inedible part in the past. Our study, which elucidated its nutritional value, supported the dietary recommendations (40) proposed by nutrients that increasing whole grain intake brings great public health benefits. The present study and previous studies proved that BPIS has potential development prospects. Therefore, on the one hand, BPIS can be used as an adjuvant formulation of current IBD therapeutic drugs, which can reduce the side effects of conventional drugs and discount the substantial economic burden on patients. On the other hand, for some groups, such as patients with colitis, patients with intestinal dysfunction, or patients with intestinal dysbiosis, BPIS can be developed as a special medical food or dietary supplement for daily consumption.

In conclusion, in DSS-induced experimental colitis, administration of BPIS suppressed the secretion of inflammatory cytokines and enhanced the functions of tight junctions, goblet cells, and mucin family. BPIS restored the gut microbiota composition and increased the contents of SCFAs, including acetic acid, propionic acid, and butyric acid. These findings highlight the developed potential of BPIS as a safe and efficient agent adjuvant for IBD improvement.

## Data availability statement

The data presented in the study are deposited in the National Center for Biotechnology Information (NCBI) repository, accession number is PRJNA855543.

## Ethics statement

The animal study was reviewed and approved by Committee on the Ethics of Animal Experiments of Shanxi University.

## Author contributions

RY: conceptualization, methodology, visualization, and writing–original draft. SS: conceptualization, data curation, supervision, and writing–review and editing. NA: methodology. FL: supervision. KC: validation. JS: visualization. HL: methodology and supervision. ZL: conceptualization, methodology, supervision, and funding acquisition. All authors contributed to the article and approved the submitted version.

## Funding

This research was funded by the National Natural Science Foundation of China (No. 32072220) and the Project of the Central Government Guiding Local Science and Technology (YDZX20201400001436). The authors also appreciate the support from the Key Laboratory of Chemical Biology and Molecular Engineering of the National Ministry of Education, Shanxi University.

## Conflict of interest

The authors declare that the research was conducted in the absence of any commercial or financial relationships that could be construed as a potential conflict of interest.

## Publisher's note

All claims expressed in this article are solely those of the authors and do not necessarily represent those of their affiliated organizations, or those of the publisher, the editors and the reviewers. Any product that may be evaluated in this article, or claim that may be made by its manufacturer, is not guaranteed or endorsed by the publisher.
